# m^6^A Methylated Long Noncoding RNA *LOC339803* Regulates Intestinal Inflammatory Response

**DOI:** 10.1002/advs.202307928

**Published:** 2024-01-25

**Authors:** Ane Olazagoitia‐Garmendia, Henar Rojas‐Márquez, Maialen Sebastian‐delaCruz, Aloña Agirre‐Lizaso, Anne Ochoa, Luis Manuel Mendoza‐Gomez, Maria J Perugorria, Luis Bujanda, Alain Huerta Madrigal, Izortze Santin, Ainara Castellanos‐Rubio

**Affiliations:** ^1^ Department of Biochemistry and Molecular Biology University of the Basque Country UPV/EHU Leioa 48940 Spain; ^2^ Biobizkaia Health Research Institute Barakaldo 48903 Spain; ^3^ Department of Genetics Physical Anthropology and Animal Physiology University of the Basque Country UPV/EHU Leioa 48940 Spain; ^4^ Department of Liver and Gastrointestinal Diseases Biogipuzkoa Health Research Institute Donostia University Hospital Donostia‐San Sebastian 20014 Spain; ^5^ Department of Medicine Faculty of Medicine and Nursing University of the Basque Country UPV/EHU Donostia‐San Sebastián 20014 Spain; ^6^ CIBERehd Instituto de Salud Carlos III (ISCIII) Madrid 28029 Spain; ^7^ Department of Medicine Medicine Faculty University of the Basque Country UPV/EHU Leioa 48940 Spain; ^8^ Gastroenterology Department Hospital Universitario de Galdakao Galdakao 48960 Spain; ^9^ Centro de Investigación Biomédica en Red de Diabetes y Enfermedades Metabólicas Asociadas CIBERDEM Instituto de Salud Carlos III Madrid 28029 Spain; ^10^ Ikerbasque Basque Foundation for Science Bilbao 48011 Spain

**Keywords:** intestinal inflammation, lncRNA, m^6^A methylation, RNA therapy, SNP

## Abstract

Cytokine mediated sustained inflammation increases the risk to develop different complex chronic inflammatory diseases, but the implicated mechanisms remain unclear. Increasing evidence shows that long noncoding RNAs (lncRNAs) play key roles in the pathogenesis of inflammatory disorders, while inflammation associated variants are described to affect their function or essential RNA modifications as N^6^‐methyladenosine (m^6^A) methylation, increasing predisposition to inflammatory diseases. Here, the functional implication of the intestinal inflammation associated lncRNA *LOC339803* in the production of cytokines by intestinal epithelial cells is described. Allele‐specific m^6^A methylation is found to affect YTHDC1 mediated protein binding affinity. *LOC339803*‐YTHDC1 interaction dictates chromatin localization of *LOC339803* ultimately inducing the expression of NFκB mediated proinflammatory cytokines and contributing to the development of intestinal inflammation. These findings are confirmed using human intestinal biopsy samples from different intestinal inflammatory conditions and controls. Additionally, it is demonstrated that *LOC339803* targeting can be a useful strategy for the amelioration of intestinal inflammation in vitro and ex vivo. Overall, the results support the importance of the methylated *LOC339803* lncRNA as a mediator of intestinal inflammation, explaining genetic susceptibility and presenting this lncRNA as a potential novel therapeutic target for the treatment of inflammatory intestinal disorders.

## Graphical abstract

The increasing interest on RNA based therapeutics make long noncoding RNAs (lncRNAs) appealing targets for the development of novel treatments. Herein we describe the allele‐specific and N^6^‐methyladenosine (m^6^A) methylation dependent ability of *LOC339803* lncRNA to regulate the expression of proinflammatory cytokines in the intestine. Deregulated *LOC339803* levels in patients with intestinal inflammation and results from ex vivo *LOC339803* reduction experiments point to this lncRNA as an interesting therapeutic target for intestinal inflammatory disorders.

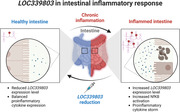

## 1 Introduction

Genetic and environmental factors have been identified to play a major role in the development of intestinal inflammatory disorders. While Genome Wide Association Studies (GWAS) and Immunochip studies have helped to identify *loci* that confer risk to these pathologies, the understanding of their underlying mechanism remains limited, mostly due to their localization in noncoding genomic regions and their individual small effect size.^[^ ^1^, ^2^ ^]^ Within the last decade, advances in RNA sequencing techniques have revealed novel noncoding RNAs, from which a high number corresponds to the family of long noncoding RNAs (lncRNAs).^[^ ^3^, ^4^ ^]^ LncRNAs are RNA molecules longer than 200 nucleotides with no or low protein coding potential. They have been involved in key cellular processes through a wide diversity of mechanisms, as they are able to bind DNA, RNA, or proteins. Indeed, lncRNAs can respond to external stimuli in a quick and cell type‐specific manner, working both transcriptionally and post‐transcriptionally.^[^ ^4^, ^5^, ^6^ ^]^ Interestingly, inflammation‐associated single nucleotide polymorphisms (SNPs) are enriched in lncRNAs ^[^ ^5^ ^]^ and some lncRNAs have been associated with different intestinal inflammatory disorders.^[^ ^7^, ^8^, ^9^ ^]^ Hence deciphering the role of inflammation associated SNPs in lncRNA function may help to understand the pathogenesis of these complex disorders.

Inflammation‐associated SNPs can also affect RNA modifications as N^6^‐methyladenosine (m^6^A).^[^ ^10^, ^11^ ^]^ m^6^A is the most abundant internal chemical modification of mRNAs and noncoding RNAs and it is involved in multiple aspects of RNA metabolism, playing crucial roles in many cellular processes. In the last years, research on m^6^A‐mediated regulatory pathways has increased exponentially ^[^ ^12^, ^13^, ^14^, ^15^ ^]^ and recent work has suggested that m^6^A may be involved in the development of intestinal pathologies.^[^ ^10^, ^16^, ^17^ ^]^ Even if m^6^A‐quantitative trait loci (QTL) have been described to be expression and splicing QTL independent,^[^ ^11^ ^]^ little is known about the genetic effects of m^6^A modification and their role in diseases. Considering that more than 85% of inflammation‐associated SNPs are in noncoding regions and their functions are difficult to assess, new approaches are needed to clear up the biological functions of noncoding SNPs. Addressing functional characterization studies of these variants by integrating m^6^A methylation data may help to identify new regulatory effects of disease‐specific associated SNPs, opening the door to the development of novel therapeutic approaches.

Chronic inflammatory diseases are a wide range of autoimmune and inflammatory diseases characterized by persistent inflammation. Specifically, intestinal inflammatory disorders are a group of diseases in which inflammation is present along the gastrointestinal (GI) tract. Having a healthy gut is important for nutrient delivery, but also to defend from infection and for proper crosstalk with peripheral and central circuits. In chronic conditions, persistent inflammation causes damage to the GI tract influencing the function of other organs.^[^ ^18^ ^]^ The most common inflammatory diseases connected to the digestive system are celiac disease (CeD) and inflammatory bowel disease (IBD), comprised of ulcerative colitis (UC) and Crohn's disease (CD). In these intestinal disorders, aberrant cytokine responses to environmental triggers (viral infections, microbiota dysbiosis, dietary agents, etc.) produced not only by the immune cells but also by non‐immune cells, such as epithelial cells, lead to chronic inflammation and destruction of healthy tissues. The sustained inflammation results in intense abdominal pain and diarrhea and it can also lead to serious intestinal and extraintestinal complications, as GI cancer or psychological symptoms.^[^ ^19^, ^20^ ^]^ Indeed, people with intestinal inflammatory disorders are at an increased risk of developing GI cancer, particularly colon cancer.^[^ ^21^, ^22^, ^23^ ^]^ Even if there are several therapeutic strategies, including different types of medication, surgery, and lifestyle changes for IBD, or a lifelong gluten free diet for CeD, there is no actual cure for these diseases, highlighting the need of more effective and specific treatments.^[^ ^24^, ^25^ ^]^

In this work, we studied the lncRNA *LOC339803*, with a previously unknown function, located on the Immunochip region 2p15. This region is associated with several organ specific inflammatory disorders including intestinal disorders.^[^ ^26^ ^]^ Interestingly, the intestinal inflammation associated SNP rs11498 is within an exon of *LOC339803* located next to a m^6^A motif, making this lncRNA an interesting candidate to study the effect of differential m^6^A methylation on lncRNA function in the context of intestinal inflammation. Here, we show that *LOC339803* presents allele‐specific m^6^A methylation levels in intestinal cells, affecting YTHDC1 mediated protein binding affinity. YTHDC1 interaction with *LOC339803* activates the nuclear factor kappa B (NFκB) key inflammatory pathway, which will ultimately cause higher basal proinflammatory cytokine levels in the individuals with the risk allele. Moreover, we also demonstrated that targeting *LOC339803* can ameliorate inflammation both in vitro and ex vivo.

## 2 Results

### 2.1 The Genotype of the Intestinal Inflammation Associated SNP rs11498 Affects m^6^A Methylation Levels and Stability of *LOC339803* lncRNA

**Figure 1:**
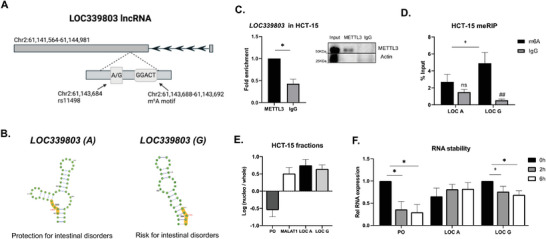
The genotype of the intestinal inflammation associated SNP rs11498 affects m^6^A methylation levels and stability of *LOC339803* lncRNA. A) Graphical representation of *LOC339803* chromosomic location created with BioRender. Immunochip SNP rs11498 and the GGACT m^6^A motif next to the SNP are zoomed in. B) Predicted allele‐specific secondary structure of the highly methylated motif region in *LOC339803* according to m^6^A predictor SRAMP online tool. C) METTL3 RNA immunoprecipitation (RIP) and quantification of bound *LOC339803* levels assessed by RT‐qPCR in HCT‐15 intestinal cells. Right, representative immunoblot of the RIP experiments with Actin as negative control for the RIP. p‐value determined by Student's t‐test. D) Allele‐specific m^6^A methylation levels of *LOC339803* assessed by RNA immunoprecipitation using anti‐m^6^A antibody in HCT‐15 intestinal cells. p‐value determined by two‐way ANOVA test. E) Subcellular localization of both *LOC339803* forms (*LOC A* and *LOC G*) using *P0* (*RPLP0*, cytoplasmic) and *MALAT1* (nuclear) as controls in HCT‐15 intestinal cells. F) RNA stability assay in HCT‐15 cells treated with actinomycin for 2 and 6 h using *RPLP0* as a positive control. p‐value determined by Student's t‐test. Data are means ± SEM (n ≥ 3 independent experiments).+*p* < 0.1, ^*^ *p* < 0.05; Enrichment relative to control IgG ##*p* < 0.01.

The SNP rs11498 (GRCh38:chr2:61143684), within the IBD‐associated Immunochip region 2p15^[^ ^27^ ^]^ is located in an exon of the uncharacterized lncRNA *LOC339803* (also known as *AC016747.3* or *C2orf74‐DT*), close to an m^6^A methylation motif (**Figure** **1**A). The Immunochip region 2p15, where the SNP is located, is associated to various inflammatory disorders including both forms of IBD, Crohn's disease and ulcerative colitis (CRO and UC respectively), and celiac disease (CEL)^[^ ^26^ ^]^ (Figure S1A, Supporting Information). Interestingly, this region presents very low conservation among vertebrates, showing no murine homolog for the lncRNA (Figure S1B, Supporting Information).

Considering that lncRNAs generally have cell‐type specific expression, we first analyzed *LOC339803* expression in RNAseq data retrieved from GTEx database.^[^ ^28^ ^]^ As observed for many other lncRNAs, *LOC339803* was differentially expressed among the analyzed tissues, showing the lowest expression levels in GI tissues as colon or small intestine and highest in certain brain regions and thyroid tissue (Figure S1C, Supporting Information). To further confirm these results, a commercially available RNA pool of different human tissues was used to quantify *LOC339803* expression by RT‐qPCR, also showing similar tissue specific expression patterns (Figure S1C, Supporting Information). The differential expression levels observed among tissues suggest that this lncRNA could have cell type specific functions contributing to the development of different inflammatory diseases. Interestingly, and supporting this idea, while the G allele confers risk for intestinal inflammation, the opposite allele, A, is the risk allele for other organ specific immune disorders such as multiple sclerosis or psoriasis. Inflammation occurs by the crosstalk of immune cells with target tissue cells, as intestinal epithelial cells, mediated by cytokines and chemokines produced by both cell types. RNAseq data from the Epigenome Roadmap project^[^ ^29^ ^]^ showed that this lncRNA is barely expressed in immune cells, but presents consiredable expression levels in other tissues as cerebellum and small intestine (Figure S1D, Supporting Information). Hence, further functional studies of the lncRNA were performed in intestinal epithelial cells.

An examination of the MetDB m^6^A database^[^ ^30^ ^]^ confirmed the existence of m^6^A peaks around the SNP (Figure S1E, Supporting Information) and using the online m^6^A predictor SRAMP^[^ ^31^ ^]^ we observed that the m^6^A motif located next to the SNP presents a high probability of methylation (Figure S1F, Supporting Information). Moreover, the predicted secondary structure of the site changes depending on the rs11498 genotype, with the G allele form showing a more accessible site (Figure 1B). To confirm these predictions, we used the HCT‐15 intestinal epithelial cell line as an in vitro model for intestinal disorders, as it is heterozygous for the SNP rs11498. METTL3 RNA immunoprecipitation (RIP) confirmed the binding of this m^6^A writer to *LOC339803* (Figure 1C), indicating that the m^6^A motif next to the associated SNP could be indeed methylated. Moreover, allele‐specific meRIP in the intestinal cell line further confirmed the methylation of the site, with the G allele being preferentially methylated (Figure 1D).

Apart from differences in expression abundance, lncRNA localization also plays a critical role in their function. Additionally, m^6^A methylation can influence methylated RNA localization.^[^ ^32^ ^]^ Subcellular localization assessment showed that both allelic forms of *LOC339803* are mainly nuclear in HCT‐15 intestinal cells (Figure 1E). When the stability of the lncRNA was studied, we could observe that while *LOC A* was quite stable, the preferentially methylated form *LOC G* significantly decreased upon incubation with actinomycin D (Figure 1F); suggesting that increased methylation could lead to a reduced stability of this nuclear lncRNA.^[^ ^33^ ^]^

Hence, we confirmed that the genotype of the intestinal inflammation associated SNP rs11498 influences m^6^A methylation levels on *LOC339803* and that this differential methylation seems to influence the stability of nuclear *LOC339803* in intestinal epithelial cells.

### 2.2 YTHDC1 m^6^A Reader Interacts with *LOC339803* Influencing its Cellular Localization and Protein Binding

**Figure 2:**
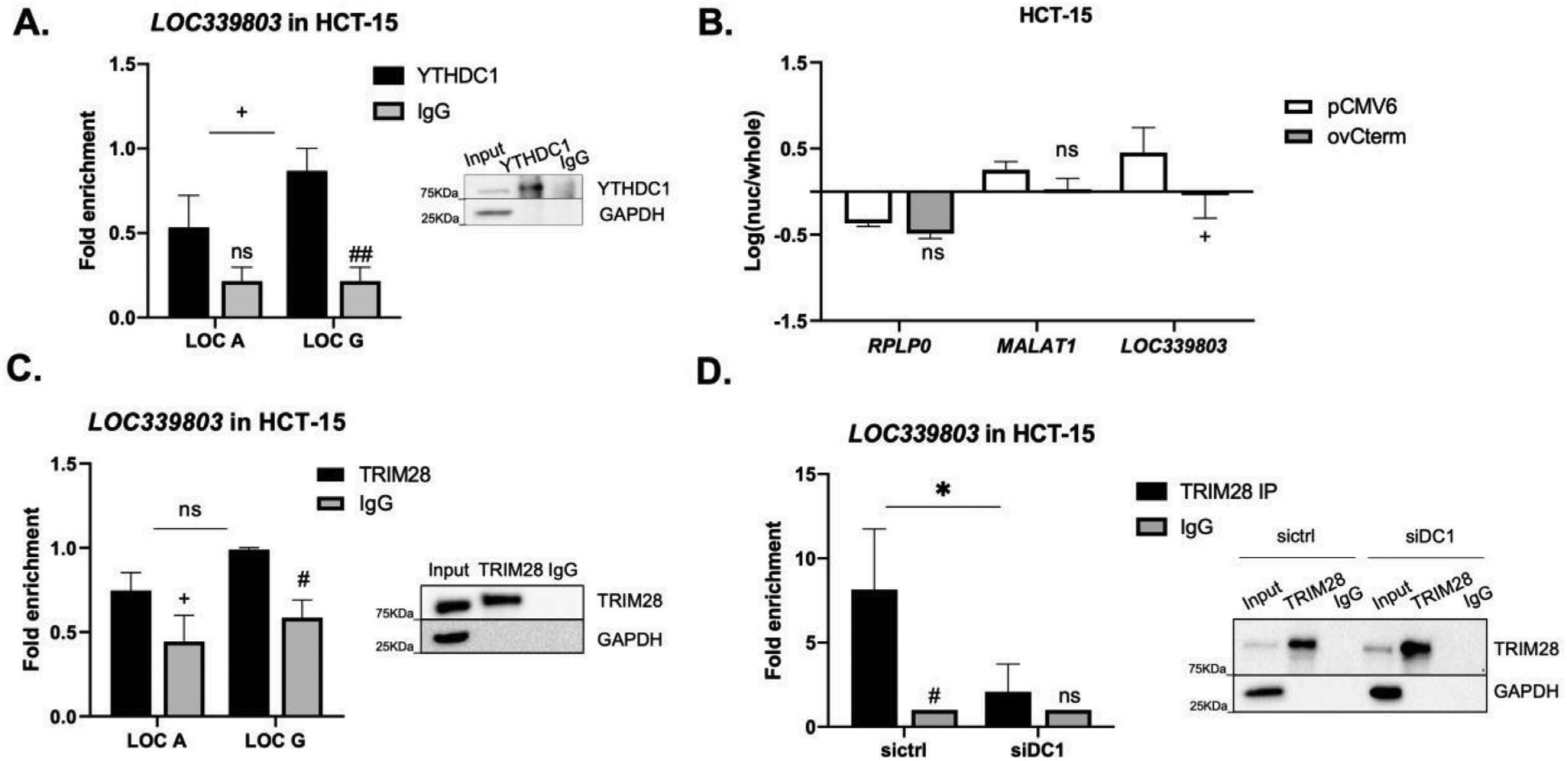
YTHDC1 m^6^A reader interacts with *LOC339803* influencing its cellular localization and protein binding. A) YTHDC1 RIP and quantification of allele‐specific *LOC339803* levels assessed by RT‐qPCR in HCT‐15 intestinal cells. Right, representative immunoblot of the RIP experiment with GAPDH as negative control for the IP. B) Subcellular localization of *LOC339803* using *RPLP0* (cytoplasmic) and *MALAT1* (nuclear) as controls upon YTHDC1 C‐terminus overexpression (ovCterm) in HCT‐15 intestinal cells. C) TRIM28 RIP and quantification of bound allele‐specific *LOC339803* levels assessed by RT‐qPCR in HCT‐15 intestinal cells. Right, representative immunoblot of the RIP experiment with GAPDH as negative control for the IP. D) TRIM28 immunoprecipitation and quantification of bound *LOC339803* levels assessed by RT‐qPCR in control (sictrl) or *YTHDC1* silenced (siDC1) HCT‐15 cells. Right, representative immunoblot of the RIP experiment with GAPDH as negative control for the IP. All p‐values determined by two‐way ANOVA test. Data are means ± SEM (*n* = 4 independent experiments). +*p* < 0.1, ^*^ *p* < 0.05, ^**^ *p* < 0.01; Enrichment relative to control IgG #*p* < 0.05, ##*p* < 0.01.

It is widely known that m^6^A methylated RNAs are recognized and bound by m^6^A readers to influence their function. The nuclear YTHDC1 m^6^A reader protein has been described to regulate a wide variety of RNA processes. More specifically, binding to the C‐terminus of YTHDC1 has been proven to affect mRNA export^[^ ^32^ ^]^ while interaction with the N‐terminus of YTHDC1 has been linked with splicing or *XIST* mediated gene repression.^[^ ^34^, ^35^ ^]^ Given that YTHDC1 influences chromatin associated RNA stability^[^ ^33^ ^]^ as well as the binding to target chromatin sites,^[^ ^34^, ^35^, ^36^ ^]^ we wondered whether this nuclear reader could be interacting with *LOC339803* in our intestinal model (Figure S2A, Supporting Information). YTHDC1 RIP confirmed its interaction with *LOC339803* (Figure S2B, Supporting Information), showing preferentially binding to the G allele (**Figure** **2**A), concordant with our previous observations (Figure 1D). Interestingly, other cytoplasmic m^6^A readers did not show interaction with our lncRNA (data not shown). We further studied the effect of YTHDC1 specific binding in the subcellular localization of *LOC339803*. We observed that overexpression of the C‐terminus in HCT‐15 cell line resulted in a redistribution of our nuclear *LOC339803* toward the cytoplasm while the overexpression of the N‐terminus or the full length YTHDC1 did not change lncRNA localization (Figure 2B; Figure S2C, Supporting Information). These results support that the interaction of *LOC339803* with the N‐terminus retains *LOC339803* within the nucleus and suggest that differential binding of the lncRNA to YTHDC1 termini could influence its localization, what will in turn regulate its function.

Given that YTHDC1 has been described to interact with nuclear lncRNAs and affect their transcriptional regulatory roles,^[^ ^33^, ^35^, ^37^ ^]^ we wanted to analyze the downstream effect of YTHDC1 and *LOC339803* interaction. Pulldown of *LOC339803* bound proteins in HCT‐15 cell line followed by mass spectrometry confirmed that *LOC339803* interacts with a wide variety of proteins (Figure S2C and Table S2, Supporting Information). Gene Ontology (GO) term analysis^[^ ^38^, ^39^, ^40^ ^]^ showed an enrichment in nucleic acid binding, transcription machinery binding, or chromatin and histone binding (Figure S2C, Supporting Information), suggesting *LOC339803* could participate in transcriptional regulation. Interestingly, pulldown‐MS results showed that nuclear *LOC339803* binds TRIM28 and HDAC1 transcriptional repressors in HCT‐15 cells. These interactions were further confirmed by RIP experiments (Figure 2C; Figure S2E, Supporting Information), showing preferential binding of risk allele G in both TRIM28 and HDAC1; implying that in the presence of the risk allele, there is an increased binding of *LOC339803* to YTHDC1 and transcriptional repressor proteins. Interestingly, these transcription repressors had been previously identified to interact with YTHDC1 and to bind methylated RNAs.^[^ ^37^ ^]^ Indeed, silencing of YTHDC1 in HCT‐15 cells (Figure S2E, Supporting Information) lead to a reduced interaction of *LOC339803* and TRIM28 (Figure 2D), indicating that YTHDC1 is essential for *LOC339803* interaction with transcription regulators in intestinal cells.

Altogether, these results demonstrate that allele‐specific differential methylation of *LOC339803* influences YTHDC1 binding ability. Additionally, we also confirmed that YTHDC1 is necessary for the binding of *LOC339803* to transcription regulators in intestinal cells, emphasizing the key role of m^6^A in the function of the lncRNA.

### 2.3 *LOC339803* Induction Promotes Transcriptional Repression of *COMMD1* Activating NFκB Proinflammatory Pathway

**Figure 3:**
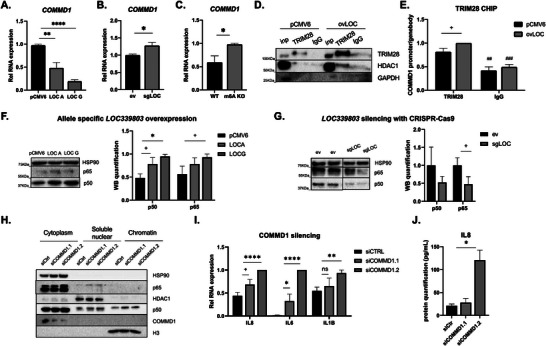
*LOC339803* induction promotes transcriptional repression of *COMMD1* activating NFkB proinflammatory pathway. Quantification of *COMMD1* RNA levels by RT‐qPCR in cells A) transfected with overexpression plasmids of both forms of *LOC339803* (*LOC A* and *LOC G*), B) with depleted *LOC339803* and C) with a deletion of the m^6^A methylation region in *LOC339803* using CRISPR‐Cas9. *p*‐values determined by Student's *t*‐test. D) Representative immunoblot of the co‐immunoprecipitation of TRIM28 and HDAC1 using anti‐TRIM28 antibody in cells transfected with an empty vector (pCMV6) or *LOC339803* overexpressing plasmids (ovLOC). GAPDH was used as a negative control for the Co‐IP. E) Chromatin immunoprecipitation (ChIP) using anti‐TRIM28 antibody and quantification of bound *COMMD1* promoter levels in cells transfected with an empty vector (pCMV6) or *LOC339803* overexpressing plasmids (ovLOC). *p* value determined by two‐way ANOVA test. Quantification of NFκB subunits p50 and p65 protein levels in cells F) transfected with overexpression plasmids of both forms of *LOC339803* (*LOC A* and *LOC G*) and G) with depleted *LOC339803* using CRISPR‐Cas9. HSP90 was used as loading control. *p*‐values determined by Student's *t*‐test. HCT‐15 cells were transfected with control siRNAs and two different siRNAs against *COMMD1*. H) NFκB activation in COMMD1 silenced cells was assessed by quantification of p50 and p65 subunits in the nuclear compartments. HSP90 was used as cytoplasmic control, HDAC1 was used as nuclear soluble control and H3 was used as chromatin bound protein control. NFκB target proinflammatory cytokines were quantified at RNA and protein levels by RT‐qPCR I) and ELISA J), respectively. p values determined by ANOVA tests. All data are means ± SEM (n ≥ 3 independent experiments). +*p* < 0.1, ^*^ *p* < 0.05, ^**^ *p* < 0.01, ^***^ *p* < 0.001, ^****^ *p* < 0.0001. Enrichment relative to control IgG ##*p* < 0.01, ###*p* < 0.001.

Having confirmed that *LOC339803* can bind transcription repressor proteins by a m^6^A dependent mechanism, we further studied *LOC339803* mechanism in intestinal cells. Assessment of its localization revealed that being primary nuclear, it was equally distributed between the nucleoplasm and the chromatin (Figure S3A, Supporting Information). Considering that nuclear lncRNAs generally act in cis, regulating the transcription of their neighboring genes, and that we had already observed that *LOC339803* binds transcription repressor proteins (Figure 2C; Figure S2D,E, Supporting Information) we resolved to analyze the expression of intestinal inflammation‐related genes located in the genomic vicinity of *LOC339803* after overexpression of the lncRNA (Figure S3B, Supporting Information). Among the analyzed genes, only *COMMD1* presented significant expression changes in response to *LOC339803* overexpression (**Figure** **3**A,C), with decreased levels that were more pronounced in the presence of the risk allele (*G). On the other end, cells with a deletion of the lncRNA showed increased *COMMD1* expression (Figure 3B; Figure S3D, Supporting Information). Accordingly, cells with a CRISPR‐Cas9 mediated deletion of the m^6^A motif region in *LOC339803* presented induced *COMMD1* expression levels (Figure 3C; Figure S3E, Supporting Information), confirming the implication of *LOC339803* and the relevance of m^6^A methylation in the regulation of *COMMD1*.

As these results pointed to a lncRNA‐mediated transcriptional regulation of *COMMD1*, we wanted to describe how *LOC339803* regulates *COMMD1* expression. We had observed that YTHDC1 is necessary for *LOC339803*‐TRIM28 interaction (Figure 2D), underlying the importance of m^6^A methylation on lncRNA function. Indeed, DNase I hypersensitivity assay in HCT‐15 cells confirmed that when the risk *LOC G* form (presenting higher methylation levels) is overexpressed, the DNase I hypersensitivity site within *COMMD1* promoter is less accessible, suggesting that the lncRNA could be contributing to a lower *COMMD1* transcription (Figure S3F, Supporting Information). In addition, TRIM28, which has been described to interact with YTHDC1 and to bind methylated RNAs, works as a scaffold protein to recruit diverse repressor proteins as histone deacetylases or H3K9 methyltransferase SETDB1.^[^ ^36^, ^41^ ^]^ Using Co‐immunoprecipitation (Co‐IP), we were able to confirm that HDAC1 protein (that also binds to *LOC339803*) (Figure S2E, Supporting Information) interacts with TRIM28 in HCT‐15 cells (Figure 3D). Moreover, overexpression of *LOC339803* revealed stronger HDAC1‐TRIM28 interaction (Figure 3D) as well as an increased binding of TRIM28 to *COMMD1* promoter as assessed by chromatin immunoprecipitation (ChIP) (Figure 3E), which is concordant with an inflammatory environment.

Interestingly, *COMMD1* repression is known to be related with NFκB activation^[^ ^42^ ^]^ which is a central player in the development of intestinal inflammatory diseases.^[^ ^43^, ^44^ ^]^ In line with this, we observed augmented amounts of NFκB p50 and p65 subunits in the cells overexpressing *LOC339803*, with a significant increase only in the presence of the risk allele (*G) (Figure 3F). On the other end, cells with a deletion of the lncRNA showed a decrease in p50 and p65 levels (Figure 3G). Collectively, these results showed that *LOC339803* binds to transcription repressor proteins, forming a transcription repressor complex that regulates *COMMD1* expression, and as a result in NFκB induction. Additionally, in the presence of risk allele G this inflammation seems to be increased. *COMMD1* silencing confirmed that reduced *COMMD1* expression activates NFκB in intestinal cells as observed by the increased levels of both p50 and p65 phosphorylated subunits in the nuclear and chromatin compartments (Figure 3H).

Considering that NFκB activation results in induced proinflammatory cytokine expression, we also quantified different NFκB target cytokines. Overexpression of *LOC339803* led to an induction of proinflammatory cytokines *IL8*, *IL6* and *IL1B*, in intestinal cells (Figure S3G, Supporting Information). In accordance with these results, cells with reduced *COMMD1* expression also showed augmented expression of these proinflammatory cytokines at RNA level (Figure 3I). Moreover, to further confirm the functional implication of *COMMD1* reduction and subsequent NFκB activation in intestinal cells, we also quantified the secreted protein expression in these supernatants. Interestingly, while the secreted IL6 and IL1B levels were too low, we observed increased secretion of IL8 in *COMMD1* silenced cells (Figure 3J).

Overall, these results show that the increased methylation present in the risk allele (*G) enhances the interaction of YTHDC1 with the lncRNA, favoring the binding of *LOC339803*‐TRIM28‐HDAC1 repressor complex to *COMMD1* promoter, finally resulting in increased NFκB and proinflammatory cytokine expression in intestinal cells.

### 2.4 *LOC339803* Expression is Increased in Inflammatory Intestinal Disorders and Emerges as a Therapeutic Target

As the in vitro results obtained in intestinal cells pointed to an implication of *LOC339803* in the pathogenesis of intestinal inflammatory disorders by boosting a proinflammatory environment, we next wanted to confirm these results using human patient samples. As IBD is one of the most common intestinal inflammatory disorders, intestinal biopsies from controls and patients with the two IBD subtypes (ulcerative colitis, UC, and Crohn's disease, Crohns) were analyzed.

**Figure 4:**
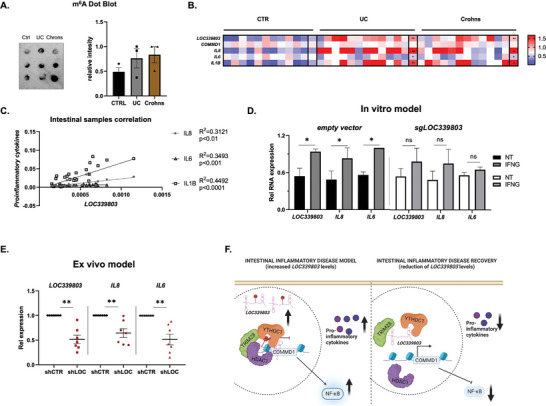
*LOC339803 expression is increased in inflammatory intestinal disorders and emerges as a therapeutic target*. A) Representative immunoblot and quantification of total m^6^A levels in intestinal biopsies from controls (CTRL) and patients with ulcerative colitis (UC) and Crohn's disease (Crohns) by dot‐blot. B) Relative RNA expression values of *LOC339803*, *COMMD1, IL8, IL6*, and *IL1B* by RT‐qPCR in intestinal biopsies from controls (CTR) and patients with ulcerative colitis (UC) and Crohn's disease (Crohns). Individual and mean values (last row of each group) of relative RNA expression represented in a heatmap. C) Correlation between proinflammatory cytokines and *LOC339803* in intestinal biopsies. r^2^ and p were calculated by Pearson correlation (*n* = 36). D) in vitro stimulation model in HCT‐15 intestinal cells using interferon gamma (IFNG). Quantification of *LOC339803* and proinflammaotry cytokines RNA levels by RT‐qPCR in cells transfected with an empty vector or *LOC339803* depleted cells using CRISPR‐Cas9 (sgLOC339803). E) ex vivo system in human intestinal biopsy samples from IBD patients presenting intestinal inflammation infected with *LOC339803* silencing lentivirus. Quantification of *LOC339803* and proinflammaotry cytokines RNA levels by RT‐qPCR. F) Representative image of stimulation and recovery models in intestinal cells created with Biorender. *p*‐values determined by Student's t‐test. Data are means ± SEM (n ≥ 3 independent experiments). ^*^ *p* < 0.05.

We first observed that IBD patients present increased total m^6^A methylation levels (**Figure** **4**A) and altered expression of several m^6^A machinery members (Figure S4A, Supporting Information), pointing to m^6^A methylation as an important player in disease related regulatory pathways.^[^ ^17^, ^45^ ^]^ Specifically, we observed that the expression of YTHDC1 reader is significantly increased in IBD patients. According to our in vitro results, this could enhance the binding of *LOC339803* and the repressor complex to *COMMD1* promoter, inducing an NFκB mediated proinflammatory environment in these individuals. Indeed, IBD patients, mainly those with UC, also present significantly increased *LOC339803* expression and a trend toward reduction of *COMMD1* levels (Figure 4B). Moreover, we also confirmed that IBD patients present increased NFκB regulated cytokine expression (Figure 4B), as a sign of increased proinflammatory environment in their intestines. Additionally, we could also corroborate that the increased *LOC339803* levels significantly correlate with higher cytokine expression in human intestinal samples (Figure 4C). To further study *LOC339803* involvement in IBD, we analyzed online expression data from induced human intestinal organoids.^[^ ^46^ ^]^ In accordance with our in vitro and human biopsy sample results, *LOC339803* expression is significantly increased in UC derived intestinal organoids compared to normal mucosa (Figure S4B, Supporting Information), which also present increased *IL8* levels. Altogether, our results in IBD patients confirm that the increased inflammation present in these individuals is, at least in part, mediated by *LOC339803*‐induced proinflammatory cytokine levels.

m^6^A methylation has also been related to other intestinal disorders as celiac disease or GI cancers.^[^ ^14^, ^47^, ^48^, ^49^, ^50^ ^]^ Moreover, it is known that chronic inflammation increases risk to develop GI malignancies.^[^ ^22^ ^]^ Interestingly, when we analyzed biopsies from CeD patients, we could also observe increased levels of total m^6^A and *LOC339803* as well as the expression of the proinflammatory cytokines (Figure S4C,D, Supporting Information). Moreover, analysis of online data from Gent2 database^[^ ^51^ ^]^ showed that *LOC339803* and the analyzed proinflammaotry cytokines are significantly overexpressed in tumor tissues from intestinal samples when compared to normal tissue expression levels (Figure S4E, Supporting Information). Lastly, data from GEPIA web app^[^ ^52^ ^]^ showed that individuals with increased *LOC339803* (*AC016747.3*) levels have lower overall survival when suffering from GI cancers (Figure S4F, Supporting Information). These results highlight the importance of a controlled regulation of the *LOC339803*‐induced inflammatory pathway to protect against the increased inflammation in patients with intestinal disorders as IBD or CeD, what could in turn derive in GI cancers and contribute to a reduced survival.

As these results in human samples confirmed the implication of *LOC339803* in the development of intestinal inflammatory pathologies, and considering the recent development on m^6^A‐targeting drugs and RNA‐based therapies, we wanted to analyze the putative therapeutic use of *LOC339803* and/or its methylation levels in an intestinal inflammatory disease model. To this aim, we first confirmed that by manipulating m^6^A machinery (overexpressing *METTL3*) or reducing total m^6^A levels using cycloleucine, *LOC339803* expression levels can be modulated (Figure S4G,H, Supporting Information). These results increase the importance of m^6^A methylation in *LOC339803* functionality as well as its significance as a potential therapeutic target and highlight m^6^A‐targeted drugs as a good approach to regulate *LOC339803* levels in intestinal inflammatory disorders as IBD.

Moreover, we developed an in vitro disease model to address the role of *LOC339803* in the regulation of proinflammatory cytokines in a disease scenario. To mimic the effect of *LOC339803* in the activation of a proinflammatory response in IBD, we used an interferon gamma (IFNG) stimulation model, described to alter m^6^A methylation levels in intestinal cells.^[^ ^45^ ^]^ With the aim of reducing IFNG‐induced inflammation in these cells, we knocked down *LOC339803* by CRISPR‐Cas9 in HCT‐15 cells (Figure 4D). We observed that *LOC339803* is augmented upon IFNG stimulation, increasing the expression of different proinflammatory cytokines in intestinal cells. Of note, the decrease of lncRNA expression by CRISPR‐Cas9 ameliorates the increase of the proinflammatory cytokines upon IFNG stimulation (Figure 4D), confirming the participation of this lncRNA on the induction of inflammation in the intestine. More interestingly, we also developed an ex vivo organ culture system in which we silenced the expression of *LOC339803* using lentiviral particles in human intestinal biopsy samples from inflammed IBD patients. The ex vivo reduction of *LOC339803* expression in these human intestinal biopsy samples, showed a decrease in *LOC339803* targeted proinflammatory cytokines (Figure 4E).

All these results show that *LOC339803* and its m^6^A methylation levels are key to induce proinflammatory cytokine expression in intestinal epithelial cells. In addition, *LOC339803* evolves as an interesting therapeutic target for IBD and other intestinal inflammatory disorders, as manipulating lncRNA expression or modifying m^6^A levels seems to help prevent the pro‐inflammatory environment that would lead to disease development (Figure 4F).

To sum up, our results in human samples confirm the functional implication of *LOC339803* in IBD, but also suggest that this lncRNA could be involved in other intestinal inflammatory disorders as CeD or GI cancers. Additionally, our data suggests that *LOC339803* expression levels could be involved in cancer‐related complications in IBD patients. What is more, manipulation of *LOC339803* expression in vitro and ex vivo showed the potential use of this lncRNA as a therapeutic target for IBD and its related comorbidities.

## 3 Discussion

In this study, we have described that genotype specific m^6^A methylation provides *LOC339803* lncRNA with the ability to regulate the expression of NFκB mediated proinflammatory cytokines via its interaction with YTHDC1 and transcription repressor proteins in intestinal epithelial cells (Figure S5, Supporting Information). We have confirmed that *LOC339803* expression levels are deregulated in patients with intestinal inflammation, and we have observed that manipulating *LOC339803* expression levels, downstream proinflammatory cytokines can be regulated, emerging as an interesting therapeutic target.

We confirmed that the genotype of the rs11498 SNP influences m^6^A methylation levels on *LOC339803* and that this lncRNA shows a methylation‐dependent function. RNA modifications are emerging as a new regulatory layer, playing critical roles in RNA processing, splicing, translation, or stability,^[^ ^53^, ^54^ ^]^ all of them crucial in the development of multiple human diseases.^[^ ^55^, ^56^ ^]^ m^6^A‐QTLs are known to contribute to the risk to develop some immune disorders; being enriched within RNA‐protein binding sites, RNA structure‐changing variants, and transcriptional features.^[^ ^11^ ^]^ In addition, SNPs are enriched in lncRNAs and studies have proved that associated SNPs can alter lncRNA functions, most probably by altering secondary structures and/or their binding ability.^[^ ^5^, ^57^, ^58^ ^]^ Indeed, in accordance with previous results, we have observed that the more methylated *LOC G* form is less stable, most probably due to increased interaction with YTHDC1 reader described to alter the stability of m^6^A methylated RNAs.^[^ ^33^ ^]^ These results show how the genotype dependent m^6^A levels can alter the lncRNA function. Moreover, we described that allele‐specific differential methylation of *LOC339803* determines YTHDC1 binding ability and this interaction is necessary for the coupling of *LOC339803* to the transcription repressor complex. This way, we present a functional implication of the SNP that increases the risk to develop intestinal inflammatory disorders as IBD. Based on our results and considering that m^6^A‐QTLs seem to affect key aspects of lncRNA functionality, identification of disease associated SNPs closely located to m^6^A methylation motifs seem to be a good approach to identify crucial players in key pathological pathways.

Cell type specific mechanisms leading to the activation of proinflammatory cytokines have been described in monocytes and macrophages and the different cellular environment has been proposed as the reason of this regulatory setup.^[^ ^59^, ^60^ ^]^ While the role of immune cells in the innate immune response has been known for a long time, increasing evidence indicates the importance of endothelial and epithelial cells in the early steps of inflammatory response.^[^ ^61^ ^]^ Hence, production of cytokines by epithelial cells and their role in early response steps are important to clarify the development of inflammatory disorders. In this work, we have shown that intestinal epithelial cells express *IL8, IL6*, *and IL1B* proinflammatory cytokines, but while low or no secreted IL6 and IL1B could be detected in HCT‐15 cell line, these cells showed increased IL8 protein secretion in response to NFκB activation. The secreted IL8 will increase the proinflammatory environment and attract immune cells, increasing the risk to develop IBD or other GI malignancies.

Lately, the interest on RNA‐based therapies, mainly those based on small RNA and mRNA vaccines is increasing. LncRNAs present low expression profiles and tissue‐specificity making them appealing therapeutic targets, as low dosages should be enough for disease treatment.^[^ ^62^ ^]^ This study highlights the putative therapeutic use of *LOC339803* in intestinal inflammation. On the one hand, we showed that manipulation of the m^6^A methylation influence *LOC339803* levels in intestinal cells. Hence, m^6^A methylation as well as the nearby associated SNP region seem key for proper lncRNA function, presenting m^6^A‐targeted approaches as another interesting alternative therapy as many m^6^A‐targeted drugs are already being tested.^[^ ^50^ ^]^ On the other hand, reduction of the lncRNA helped lower the IFNG‐induced inflammatory response in intestinal cells. More interestingly, when intestinal biopsy samples from IBD patients were infected with *LOC339803* silencing lentivirus, a reduction of the proinflammatory cytokines could also be detected, further supporting the idea that this lncRNA could be an interesting therapeutic target. Therefore, small molecules that disrupt lncRNA function or antisense oligonucleotides that lower its expression seem good tools for lncRNA‐focused therapies. The different clinical trials based on lncRNA therapies already being carried out, strengthen our results as a starting point for the development of the so needed therapies for IBD treatment.

One of the main drawbacks of our work, that also applies to many others studying lncRNAs^[^ ^63^ ^]^ is the lack of a murine homolog for *LOC339803*, making it implausible to perform preclinical studies in murine disease models. Specifically, the region in which *LOC339803* is located presents a very low conservation within vertebrates. Nevertheless, we believe that the studies performed using human in vitro and ex vivo models, and the confirmation in human intestinal samples with different intestinal malignancies, make our model strong and present enough evidence to conclude that *LOC339803* is involved in disease pathogenesis and could be a good therapeutic target.

To sum up, our results show the implication of lncRNAs harboring disease associated variants and epitranscriptomic modifications, such as m^6^A RNA methylation, to activate important inflammatory response players, as NFκB regulated proinflammatory cytokines. These results highlight the significance of studies in which the functional implication of a lncRNA is described in disease scenario and emphasize the importance of lncRNA and m^6^A methylation function in the pathogenesis of intestinal inflammatory disorders opening the door to novel therapeutic alternatives.

## 4 Experimental Section

### Human Patients and Samples

In this study, human bowel biopsy samples from inflammatory bowel disease, celiac disease, and control individuals were obtained from Hospital de Galdakao‐Usansolo (Spain) and Donostia University Hospital (Spain). In individuals at high risk of undiagnosed or in already diagnosed patients, an extra biopsy specimen was obtained in a routine colonoscopy of the inflamed segment if present. None of the patients suffered from any other concomitant immunological disease. None of the controls showed intestinal inflammation at the time of the biopsy.

This study was approved by the Basque Country Clinical Research Ethics Board (CEIm‐E ref. PI2019133). All experiments were performed in accordance with relevant guidelines and regulations.

### Cell Lines and Treatments

Intestinal HCT‐15 (#91 030 712) cell line(heterozygous for rs11498 SNP) was purchased from Sigma–Aldrich (Poole, UK) and cultured in RPMI (Lonza, #12‐115F) with 10% FBS, 100 units/mL penicillin and 100 µg/mL streptomycin.

For the intestinal inflammation model, WT and CRISPR Cas9 mediated *LOC339803* deleted HCT‐15 cells were stimulated with 100 U of IFNG for 30 min.

### Study Design

For in vitro experiments, human intestinal cell line HCT‐15 (heterozygous for rs11498 SNP) was used. All the in vitro experiments were performed at least three independent times. For ex vivo system, human intestinal biopsies from IBD patients were incubated with *LOC339803* silencing lentiviral particles for 24 h. Human intestinal biopsy samples from controls or IBD and CeD patients were used for RNA extraction and expression quantification by RT‐QPCR. Detailed methods for m^6^A methylation, RNA, and protein expression analyses are provided in online Supporting Information and methods.

### LOC339803 KO Cell Generation Using CRISPR Cas9

For *LOC339803* KO cell line generation two sgRNAs flanking the lncRNA were designed and cloned in a px459 vector. HCT‐15 cells were transfected with 250 ng of each vector and selected with puromycin. After selection, clonal cell lines were generated by serial dilution. The sequences for the sgRNAs are shown in Table S1 (Supporting Information).

### Ex Vivo LOC339803 Silencing Using Lentiviral Particles

Biopsies were taken from a localized area in the transverse colon in all subjects and immediately, specimens were washed in warmed wash buffer (RPMI 1640 medium + 0.1 mM DTT) and PBS1x. Then biopsies were placed in individual wells of tissue culture plates (p96) containing 50 µL RPMI complemented (10% FBS, 8.0 mM L‐glutamine, 3 mM Na Pyruvate, 60 mM Hepes, 100 U/mL P/S, 0.3 unit/mL bovine insulin). Each specimen was infected with 100 µL viral media (100 µL empty plKO or 50 µL shLOC1 + 50 µL shLOC2) and incubated up to 24 h at 37 °C (5% CO2).

Biopsies were then collected in 350 µL RA1 lysis buffer with 3.5 µL β‐mercaptoethanol (Macherey Nagel, #740 984.50) and kept at −80 °C until RNA extraction.

### m^6^A RNA Immunoprecipitation

of precleared RNA per sample were fragmented with RNA fragmentation buffer (100 mM Tris, 2 mM MgCl_2_) for 3 min at 95 °C and placed on ice immediately after heating. 10% of RNA was kept as input. One microgram of m^6^A antibody (Abcam, #ab151230) and control antibody (IgG, Santa Cruz Biotechnologies, Dallas, USA, #sc‐2025) were coupled to agarose A beads (GE Healthcare, Chicago, USA) in a rotation wheel for 1 h at 4 °C. After incubation, beads were washed twice in reaction buffer (150 mM NaCl, 10 mM Tris‐HCl, 0.1% NP‐40). RNA was added to the antibody‐coupled beads and incubated for 3 h at 4 °C in a rotating wheel. Subsequently, beads were washed 3X in reaction buffer, 3X in low salt buffer (50 mM NaCl, 10 mM TrisHCl, and 0.1% NP‐40), and 3X in high salt buffer (500 mM NaCl, 10 mM TrisHCl, and 0.1% NP‐40). After the last wash, beads were resuspended in Lysis buffer and RNA was extracted using the PureLink RNA extraction kit (Invitrogen, Carlsbad, USA, #12 183 016).

### RNA Immunoprecipitation Assay Followed with Mass Spectrometry (RIP‐MS)

For RIP‐MS experiments, sense and antisense *LOC339803* were amplified from cDNA using a T7 promoter primer. The PCR product was purified and used for in vitro transcribing biotinylated RNA using the T7 polymerase (Takara) and RNA biotin labeling kit (Roche). One microgram of purified *LOC339803* RNA was mixed and incubated with cell extracts from HCT‐15 cells. Streptavidin beads were added to the reaction and further incubated. After incubation, beads were washed five times. Samples were sent for mass spectrometry and subjected to in‐solution digestion followed by nano LC‐MS/MS analysis. List of RIP‐MS results is in Table S2 (Supporting Information).

### Statistical Analysis

All the statistical analyses were performed using GraphPad Prism 8 (GraphPad Software). Significance was calculated using Student's *t*‐test, Mann Whitney test, or ANOVA test as specified in figure legends. The statistical significance level was set at *p* < 0.05. *p*‐values <0.1 are marked with a +.

## Conflict of Interest

The authors declare no conflict of interest.

## Supporting information

Supporting Information

Supplemental Table 1

## Data Availability

The data that support the findings of this study are available in the supplementary material of this article.
