# Supplementary material for: m6A Methylated Long Noncoding RNA LOC339803 Regulates Intestinal Inflammatory Response
Source: Adv Sci (Weinh). 2024 Jan 25;11(13):2307928. doi: 10.1002/advs.202307928 (PMC10987157; doi:10.1002/advs.202307928)
Supplement: Supplementary file 1 — Supporting Information [file ADVS-11-2307928-s002.pdf]

ADVANCED SCIENCE
Open Access

Supporting Information

for *Adv. Sci.*, DOI 10.1002/advs.202307928

m^{6}A Methylated Long Noncoding RNA *LOC339803* Regulates Intestinal Inflammatory Response

Ane Olazagoitia-Garmendia, Henar Rojas-Márquez, Maialen Sebastian-delaCruz, Aloña Agirre-Lizaso, Anne Ochoa, Luis Manuel Mendoza-Gomez, Maria J Perugorria, Luis Bujanda, Alain Huerta Madrigal, Izortze Santin and Ainara Castellanos-Rubio**

Supporting Information

m^{6}A methylated long noncoding RNA *LOC339803* regulates intestinal inflammatory response

Ane Olazagoitia-Garmendia, Henar Rojas-Márquez, Maialen Sebastian-delaCruz, Aloña Agirre-Lizaso, Anne Ochoa, Luis Manuel Mendoza-Gomez, Maria J Perugorria, Luis Bujanda, Alain Huerta Madrigal, Izortze Santin and Ainara Castellanos-Rubio**

1. SUPPLEMENTARY METHODS

DNA, RNA and protein extraction : For human biopsies NucleoSpin TriPrep kit (Macherey-Nagel, Düren, Germany, #740966.50) was used following manufacturer instructions. For HCT-15 RNA extraction was performed using NucleoSpin RNA Kit (Macherey Nagel, #740984.50) and cells were lysed in RIPA buffer for protein quantification.

Gene expression analyses: 500-1000 ng of RNA were used for the retrotranscription reaction using iScript cDNA Synthesis Kit (BioRad, CA, USA, #1708890). Expression values were determined by RT-qPCR using Sybr Green (iTaq SYBR Green Supermix, Bio-Rad, #1725124) and specific primers. Allele-specific expression was determined by RT-qPCR using a custom Taqman Genotyping Assay. *RPLP0* gene was used as endogenous control in both human samples and cell lines. Reactions were run in a BioRad CFX384 and melting curves were analyzed to ensure the amplification of a single product. All qPCR measurements were performed in duplicates and expression levels were analyzed using the $2^{-\Delta\Delta Ct}$ method and normalization to the highest value was used for relative RNA expression calculation. All primers are listed in Table S1.

SNP genotyping: Genotyping of the SNP rs11498 was performed in DNA samples of human biopsies using a custom Taqman SNP Genotyping Assay (ThermoFisher, Waltham, MA) following the manufacturer's instructions.

Western Blot: Laemmli buffer (62 mM Tris-HCl, 100 mM dithiothreitol (DTT), 10 % glycerol, 2 % SDS, 0.2 mg/ml bromophenol blue, 5 % 2-mercaptoethanol) was added to the protein extracts in RIPA and were denatured by heat. Proteins were migrated on 10% SDS-PAGE gels. Following electrophoresis, proteins were transferred onto nitrocellulose membranes using a Transblot-Turbo Transfer System (Biorad) and blocked in 5 % non-fatty milk diluted in TBST (20 mM Tris, 150 mM NaCl and 0.1 % Tween 20) at room temperature for 1 h. The membranes were incubated overnight at 4ºC with primary antibodies diluted 1:1000 in TBST. Immunoreactive bands were revealed using the Clarity Max ECL Substrate (BioRad, #1705062) after incubation with a horseradish peroxidase-conjugated anti-mouse or anti-rabbit (1:10000 dilution in 2.5 % non-fatty milk) secondary antibody for 1 h at room temperature. The immunoreactive bands were detected using a Bio-Rad Molecular Imager ChemiDoc XRS and quantified using the ImageJ software. The following antibodies were used for Western Blotting: METTL3 (Abcam, #195352), ACTIN (Santa Cruz Biotechnologies, #sc47778), YTHDC1 (Abcam, #264375, Cell Signalling, #E4I9E), GAPDH (Santa Cruz Biotechnologies, #sc-47724), TRIM28 (Santa Cruz Biotechnologies, #sc-515790), HDAC1 (proteintech, #66085-1), HSP90 (Cell Signaling; #4874), p50 (Santa Cruz Biotechnologies, #sc-8414), p65 (Santa Cruz Biotechnologies, # sc-372), H3 (Santa Cruz Biotechnologies, #sc-517576), COMMD1 (Abcam, #ab224727).

ELISA: HCT-15 cell culture supernatants were collected for determination of secreted IL8, IL6 and IL1B cytokine levels using commercially available ELISA kit (R&D Systems, Abingdon, UK; #D8000C; #DY206-05; #DY401-05) following the manufacturer's instructions.

Plasmid construction: LOC339803 was amplified from human cDNAs containing A or G allele for rs11498 SNP and cloned into a pCMV6 vector (Origene, #PS100001) using KpnI and FseI restriction sites.

For YTHDC1 C-term construct the YTH domain together with the C-terminal were cloned in a CMV driven vector using AscI and FseI restriction sites. The primers used for cloning are listed in Table S1.

pLKO.1-TRC Cloning vector (Addgene, #10878) was used for the construction of plKO.1-shLOC339803.1 and plKO.1-shLOC339803.2 plasmids. Following Addgene's protocol shRNAs were designed to knock down human *LOC339803*. The sequences for the oligos are shown in Table S1.

Overexpression: For C-termini overexpression experiments 400 ng plasmid were used. 300.000 cells/well were seeded and transfected using X-TremeGENE HP DNA transfection reagent (Sigma-Aldrich, #6366546001), cells were harvested after 48 h.

For *LOC339803* overexpression, 200 ng of plasmids per 100.000 cells were used. Cells were seeded and transfection was performed with X-TremeGENE HP DNA transfection reagent (Sigma-Aldrich, #6366546001) for 24 h.

Silencing experiments: For *YTHDC1* or *COMMD1* silencing, 30 nM of 2 different siRNAs against *YTHDC1* (IDT, #hs.Ri.YTHDC1.13.1 and hs.Ri.YTHDC1.13.3), *COMMD1* (IDT, #hs.Ri.COMMD1.13.2 and hs.Ri.COMMD1.13.3) or negative control siRNA (IDT #51-01-14-01) were transfected into cells using Lipofectamine RNAimax reagent (Invitrogen).

For *LOC339803* silencing in biopsies, viral particles were produced in HEK293FT cells transfected with 1 μg pLKO.1 shRNA plasmid, 750 ng psPAX2 packaging plasmid (Addgene, #12260) and 250 ng pMD2.G envelope plasmid (Addgene, #12259) using X-TremeGENE HP DNA transfection reagent (Sigma-Aldrich, #6366244001) in DMEM without antibiotics and cells were incubated o/n at 37ºC. Transfection media was replaced with fresh complete DMEM and viral particle containing media was harvested after 24 h and 48 h. Collected media was centrifuged and concentrated 10 times using Lenti-X concentrator (Takara, #631232). Viral

particles were stored in aliquots at -80ºC until used. Human intestinal biopsy samples were infected with sh-LOC339803 or plKO.1 as negative control for 24h.

m⁶A KO cell generation using CRISPR Cas9: For m^6A KO cell line generation, two sgRNAs flanking the m^6A motif were designed and cloned in px458 GFP and px330 mCherry vectors. HCT-15 cells were transfected with 250 ng of each plasmid. HCT-15 cells were sorted by cell sorter BD FACSJazz (2B/4YG) 48 hours post-transfection for the generation of clonal cell lines. The sequences for the sgRNAs are shown in Table S1.

RNA immunoprecipitation assay (RIP): For RIP experiments, HCT-15 cells were lysed in RIP buffer (150 mM KCl, 25 mM Tris, 0.5 mM DTT, 0.5 % NP-40, PI), kept on ice for 15 minutes and homogenized using a syringe. Lysates were pre-cleared with protein A-Agarose beads (GE Healthcare, Chicago, USA) for 1 h in a wheel shaker at 4ºC. A-Agarose beads were blocked with 20 % BSA and mixed with pre-cleared lysates and 1 µg of anti-IgG antibody (negative control; Santa Cruz Biotechnologies, #sc-2025) or antibody of interest. After overnight incubation in a wheel shaker at 4ºC, beads were washed 3X with RIP buffer, 3X with low salt buffer (50 mM NaCl, 10 mM Tris-HCl, 0.1 % NP-40) and 3X with high salt buffer (500 mM NaCl, 10 mM Tris-HCl, 0.1 % NP-40). After the washes, 70 % of beads were resuspended in RNA extraction buffer and 30 % was used for WB.

Co-immunoprecipitation assay (CO-IP): For CO-IP experiments, HCT-15 cells were lysed in CO-IP buffer (20 mM Tris pH8, 150 mM NaCl, 1mM EDTA, 1mM EGTA, 1% Triton X-100) and the same protocol described for RIP was followed. TRIM28 (Santa Cruz Biotechnologies, #sc-515790) antibody was used for CO-IPs.

Chromatin immunoprecipitation assay (ChIP): For ChIP experiments, HCT-15 cells were crosslinked with formaldehyde and collected in PBS with a scratcher. Cell pellet was then resuspended in L1 buffer (50 mM Tris pH8, 2 mM EDTA, 0.1 % NP-40, 10 % glycerol) + PI and incubated in ice for 5 min. Supernatant was discarded and pellet resuspended in 300 uL L2

Buffer (50 mM Tris pH8, 0.1 % SDS, 5 mM EDTA) + PI to disrupt the chromatin using bioruptor sonicator. Centrifuged samples at maximum speed were used for immunoprecipitation.

ChIP dilution buffer (50 mM Tris pH8, 0.5 % NP-40, 0.2 M NaCl, 0.5 mM EDTA) was added up to 1 mL and in order to reduce non-specific background, the samples were pre-incubated with 60 uL of protein A-Agarose beads (GE Healthcare, Chicago, USA) + Salmon Sperm DNA (Invitrogen #15632-011) (1 ug of DNA/20 uL of protA) for 60 mins at 4°C shaking. The supernatant was collected and equal volumes were put into 2 tubes.

A negative control antibody (IgG, Santa Cruz Biotechnologies, #sc-2025) or the antibody of interest (TRIM28, Santa Cruz Biotechnologies, #sc-515790) and 60 uL of blocked protein A-Agarose beads (with 10% BSA) were added to lysate and incubated overnight at 4°C on a shaker. After overnight incubation, beads were washed 3X with high salt wash buffer (20 mM Tris pH8, 0.1 % SDS, 1 % NP-40, 2 mM EDTA, 0.5 M NaCl) and 3X with TE buffer (10X TE buffer: 0.1 M Tris HCl, 0.001 M EDTA pH8). After the washes, the beads were resuspended in NTI buffer for DNA purification using NucleoSpin Gel and PCR Clean-up kit (Macherey-Nagel, #740609.250).

Cellular fractionation: For the quantification of RNA amounts in nuclear and cytoplasmic compartments, nuclei were isolated using C1 lysis buffer (1.28 M sucrose, 40 mM Tris-HCl pH 7.5, 20 mM $MgCl_2$, 4 % Triton X-100). The amounts of *LOC339803-A/G*, *MALAT1* (nuclear control) and *RPLP0* (cytoplasmic control) were measured by RT-qPCR and compared to the total amount of those RNAs in the whole cell lysate.

For the quantification of RNA amounts in chromatin and nucleoplasm compartments, HCT-15 cells were crosslinked using 16% formaldehyde. After crosslinking, cells were centrifuged and resuspended in NARA buffer (500 mM HEPES pH7.9, 1 M KCl, 500 mM EDTA, 0.05 % NP-40) for cytoplasm separation. Nuclei were resuspended in low-salt buffer (10 mM Tris HCl pH

7.4, 0.2 mM $MgCl_2$, 1 % triton) and after centrifugation nucleoplasm was transferred to fresh tubes. Chromatin was resuspended in HCl 0.2 N centrifuged and neutralized with 1 M Tris-HCl pH8. Obtained lysates were decrosslink prior to RNA extraction.

For the quantification of protein amounts in nuclear and cytoplasmic compartments, cells were resuspended in NARA buffer (10 mM HEPES pH 7.9, 10 mM KCl, 0.1 mM EDTA) with PI and incubated in ice for 10 minutes. After adding NP-40 to final concentration 0.05 %, lysates were incubated 5 minutes in ice and centrifuged at 400 g for 2 minutes. The supernatant was the cytosolic fraction. Pellet was washed 3X with NARA buffer and resuspended in NARC buffer (20 mM HEPES, 400 mM NaCl, 1 mM EDTA) + PI, shaken at 4ºC for 30 minutes and centrifuged at 16.000 g for 10 minutes. The supernatant was the nuclear extract.

DNase I Hypersensitivity Assay: $1x10^6$ cells per condition were transfected with pCMV6 and both *LOC339803* overexpression plasmids. Then cells were washed and collected with cold PBS. Cells were resuspended in C1 lysis buffer (1.28 M sucrose, 40 mM Tris-HCl pH 7.5, 20 mM $MgCl_2$, 4 % Triton X-100) and incubated in ice for 15min. Nuclei were pelleted, resuspended in Nuclei wash buffer (10mM Tris pH 7.4, 60mM KCl, 15mM NaCl, 5mM $MgCl_2$, 30mM sucrose) and separated in two tubes. One of the tube was incubated with DNase I at 37ºC for 30min and the other was left untreated as a negative control. DNA was extracted and same amount of DNA was used to quantify by RT-QPCR. Used primers are listed in Table S1.

Dotblot: 200 ng of RNA was crosslinked into a nitrocellulose membrane using UV and blocked using 5 % milk in 0.1 % Tween in PBS. Membrane was incubated overnight with a m^6A antibody (1:200) (Abcam, Cambridge, UK, #ab151230) at 4ºC. After washing in 0.1 % PBST, membranes were incubated with a secondary HRP- conjugated anti-rabbit antibody (1:10000) (Santa Cruz Biotechnology, #sc-2357) and the membrane was developed using Clarity Max ECL Substrate (BioRad, #1705062).

Bioinformatic packages: UCSC Multiz Alignments of 30 Vertebrates track was used in UCSC genome browser (**http://genome.ucsc.edu**)[1] to study the evolutionary conservation of *LOC339803* locus in Rhesus, mouse, dog, elephant, chicken, X_tropicalis and zebrafish species.

WashU Epigenome Browser[2] provides visualization, integration and analysis tools for epigenomic datasets. WashU Epigenome Browser v46.2 was used to visualize RNAseq counts from Epigenome Roadmap project[3] of *LOC339803* gene in different cells types.

MeT-DB V2.0 m^6A database[4] was used for assessing the existence of m^6A peaks in *LOC3399803*. MeT-DB V2.0 records predicted transcriptome-wide m^6A peaks and single-base m^6A sites from a significantly expanded collection of Methylated RNA Immunoprecipitation Sequencing (MeRIP-Seq) samples. It provides a genome browser to help visualize the m^6A sites from different studies.

SRAMP (sequence-based N^6-methyladenosine (m^6A) modification site predictor)[5] was used to predict m^6A modification sites on the allele specific RNA sequences of LOC339803 and for secondary structure prediction.

GO MOLECULAR FUNCTION analysis[6–8] was used with *LOC339803* bound nuclear proteins in HCT-15 intestinal cells from our RIP-MS and p-value results were illustrated using GraphPad Prism 8 (GraphPad Software).

GENT2 (Gene Expression database of Normal and Tumor tissues 2)[9] is an updated version of GENT, a user friendly platform gathering gene expression from diverse normal and tumor tissues from public data sets. It was used to analyze the expression profile of *LOC339803* and the proinflammatory cytokines in different intestinal tumors.

GEPIA (Gene Expression Profiling Interactive Analysis)[10] is an interactive web server for analyzing the RNA sequencing expression data of tumors and normal samples from the TCGA

and the GTEx projects. It was used to study the overall survival and expression profiles in GI cancers using *survival analysis* and *boxplots* functions.

2. SUPPLEMETARY FIGURES

Figure S1. The genotype of the intestinal inflammation associated SNP rs11498 affects m^{6}A methylation levels and stability of *LOC339803* lncRNA

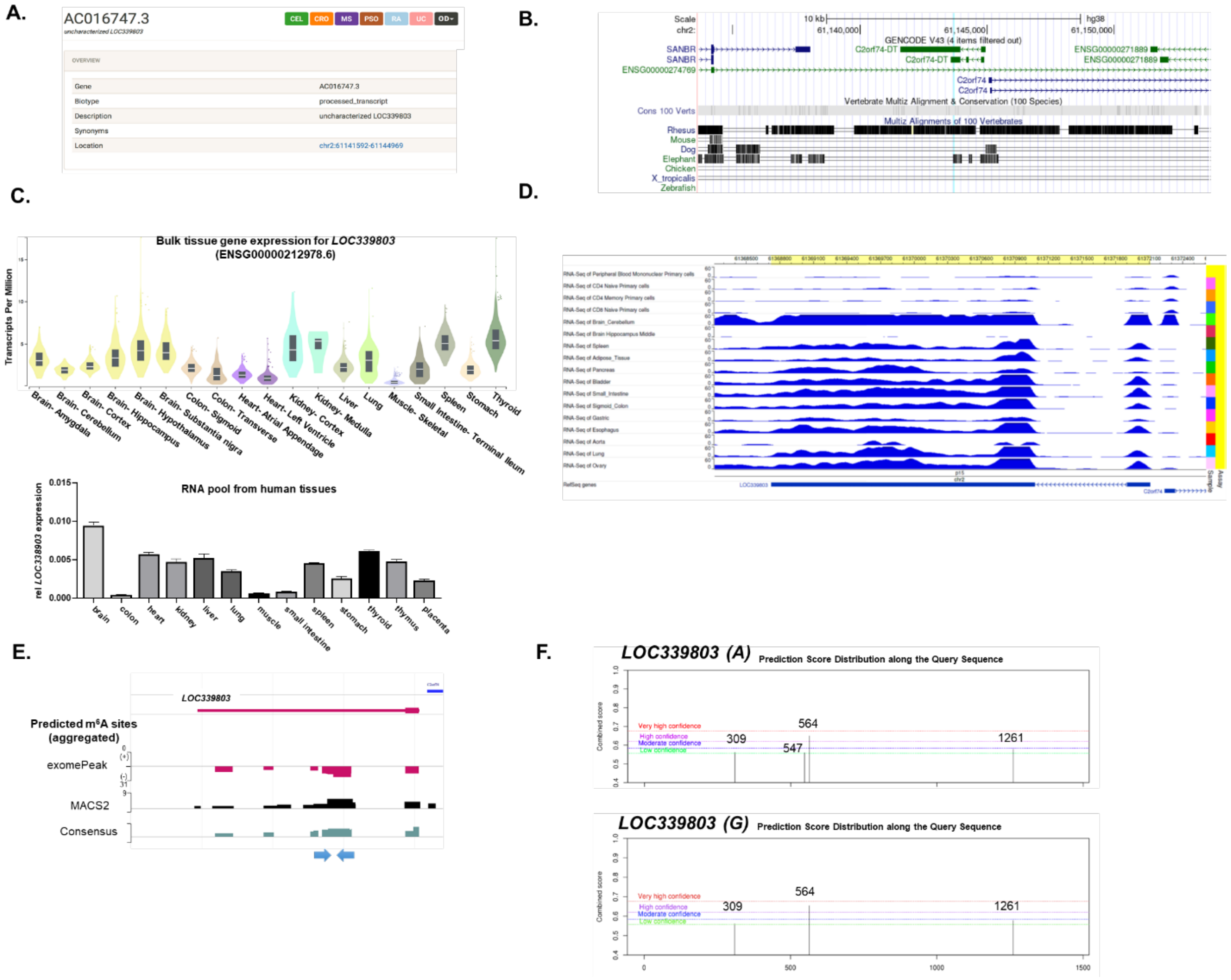

(A) *LOC339803* (also known as AC016747.3) association to celiac disease (CEL), Chrons' disease (CRO), multiple sclerosis (MS), psoriasis (PSO), rheumatoid arthritis (RA), ulcerative colitis (UC) according to Immunobase database (https://genetics.opentargets.org/immunobase). **(B)** Conservation analysis among different species of the studied region using UCSC Genome Browser (https://genome-euro.ucsc.edu/cgi-bin/hgGateway?redirect=manual&source=genome.ucsc.edu) (*LOC339803* is named as C2orf74-DT). Blue line corresponds to rs11498 SNP. **(C)** Up, *LOC339803* RNAseq data from GTEx database (https://gtexportal.org/home/), and down, relative expression of *LOC339803* in commercially available RNA pool of different human tissues. **(D)** LOC339803 RNAseq data from Epigenome Roadmap database (http://epigenomegateway.wustl.edu/legacy/). **(E)** m^{6}A peaks in *LOC339803* according to MetDB m^{6}A database. Arrows indicate the position of the meRIP primers (http://compgenomics.utsa.edu/MeTDB/). **(F)**

Allele-specific predicted m^{6}A methylation in *LOC339803* according to m^{6}A predictor SRAMP online tool (https://www.cuilab.cn/sramp).

Figure S2. YTHDC1 m^{6}A reader interacts with *LOC339803* influencing its cellular localization and protein binding.

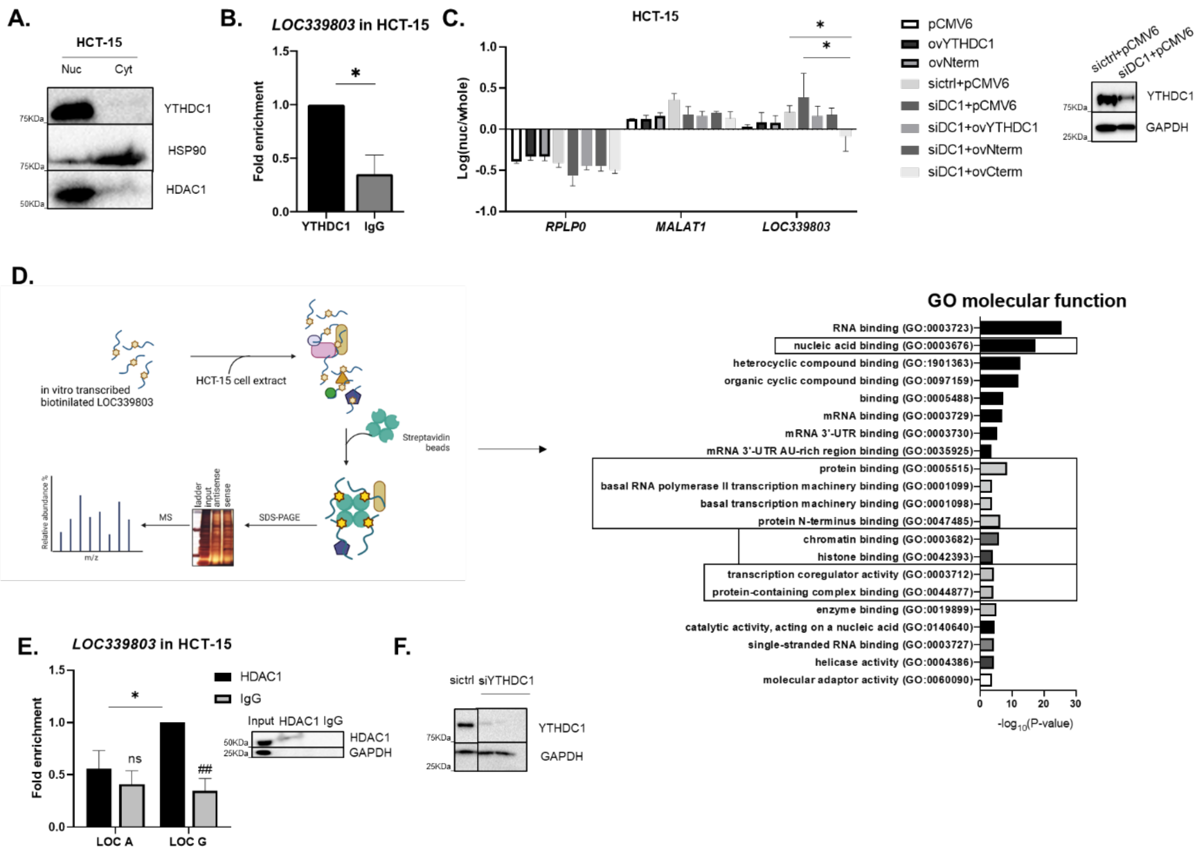

(A) YTHDC1 protein quantification by western blot in nuclear and cytoplasmic fractions in HCT-15 intestinal cells. HSP90 (cytoplasmic) and HDAC1 (nuclear) were used as controls. **(B)** YTHDC1 immunoprecipitation and quantification of bound *LOC339803* levels assessed by RT-qPCR in HCT-15 intestinal cells. Data are means ± SEM (n=3 independent experiments). p-values determined by Student's t-test. **(C)** Left, *LOC339803* subcellular localization in YTHDC1 manipulated cells using *RPLP0* (cytoplasmic) and *MALAT1* (nuclear) as controls. Empty vector plasmid (pCMV6), whole YTHDC1 construct (ovYTHDC1) or N-terminal of YTHDC1 (ovNterm) were overexpressed in HCT-15 basal cells. The different YTHDC1 constructs, whole YTHDC1 (ovYTHDC1), N-terminal (ovNTERM) or C-terminal (ovCTERM), were also overexpressed in YTHDC1 silenced cells (siDC1). YTHDC1 silencing (siDC1+pCMV6) was confirmed compared to siRNA control (sictrl+pCMV6). Right, representative immunoblot of YTHDF1 silencing. GAPDH was used as loading control. p-values determined by two-way ANOVA test. **(D)** Left, graphical representation of performed protocol for RIP-MS created with BioRender. Right, GO molecular function enrichment analysis in *LOC339803* binding nuclear proteins from RIP-MS in HCT-15 cells. **(E)** HDAC1 RIP and allele specific quantification of bound *LOC339803* levels assessed by

RT-qPCR in HCT-15 intestinal cells. Representative immunoblot of the RIP experiment with GAPDH as negative control for the IP. Data are means ± SEM (n=3 independent experiments). p-values determined by two-way ANOVA test. **(F)** Representative immunoblot of *YTHDC1* silencing. GAPDH was used as loading control. *p<0.05. Enrichment relative to control IgG ##p<0.01

Figure S3. *LOC339803* induction promotes transcriptional repression of *COMMD1* activating NFκB proinflammatory pathway

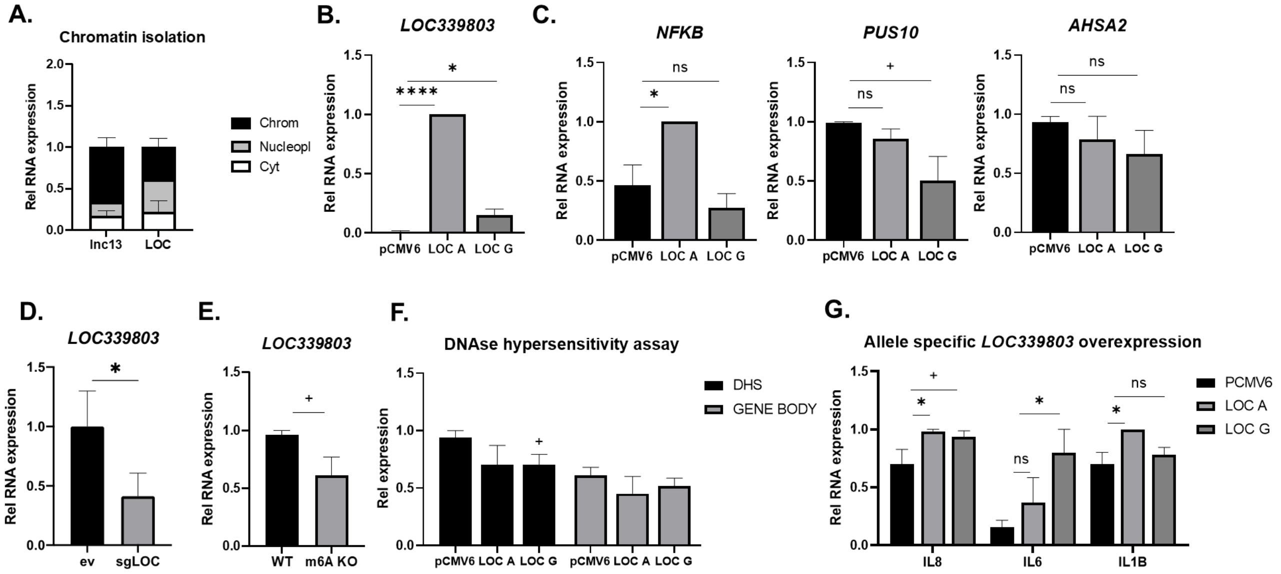

(A) Subcellular localization of *LOC339803* using *lnc13* lncRNA (chromatin associated) as control in cytoplasmic, nucleoplasm and chromatin fractions from HCT-15 intestinal cells. Data are means ± SEM (n=3 independent experiments). Quantification of *LOC339803* **(B)** and **(C)** *NFKB, PUS10* and *AHSA2* RNA levels by RT-qPCR in cells transfected with overexpression plasmids of both forms of *LOC339803* (*LOC A* and *LOC G*). Data are means ± SEM (n=3 independent experiments). p-values determined by Student's t-test. **(D)** Quantification of *LOC339803* RNA levels by RT-qPCR in cells with depleted *LOC339803* using CRISPR-Cas9. Data are means ± SEM (n≥5 independent experiments). p-value determined by Student's t-test. **(E)** Quantification of *LOC339803* RNA levels by qPCR in cells with a deletion of the m^6A methylation region in *LOC339803* using CRISPR-Cas9. Data are means ± SEM (n=4 independent experiments). p-value determined by Student' s t-test. **(F)** Quantification of chromatin accessibility using primers flanking a DNAse I hypersensitibity site (DHS) within *COMMD1* promoter and primers targeting the gene body as control. **(G)** *IL8, IL6* and *IL1B* RNA levels by RT-qPCR in cells transfected with overexpression plasmids of both forms of *LOC339803* (*LOC A* and *LOC G*). Data are means ± SEM (n=4 independent experiments). p-values determined by Student's t-test. +p<0.1, *p<0.05, ****p<0.0001.

Figure S4. *LOC339803* expression is increased in inflammatory intestinal disorders and emerges as a therapeutic target

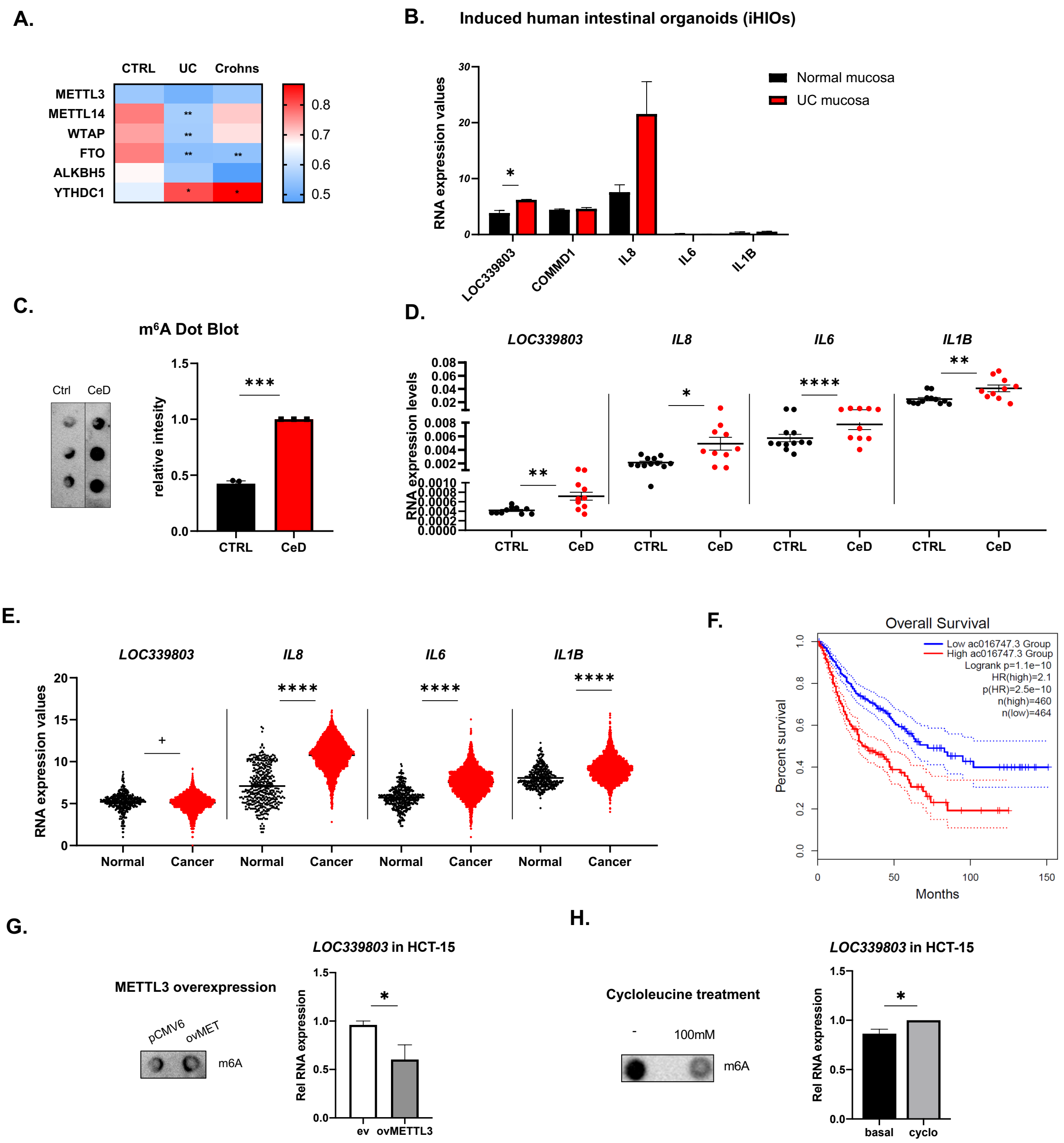

(A) Heatmap of relative expression of m^{6}A machinery genes (*METTL3*, *METTL14*, *WTAP*, *FTO*, *ALKBH5* and *YTHDC1*) quantified by RT-qPCR in intestinal biopsies from controls and patients with ulcerative colitis (UC) and Crohn's disease (Crohns). Data are represented as mean values. (n=12) **(B)** Relative RNA expression values of *LOC339803*, *COMMD1*, *IL8*, *IL6* and *IL1B* in induced human intestinal organoids from normal mucosa (n=2) and UC derived mucosa (n=3) (REF). **(C)** Quantification of total m^{6}A levels by dot-blot in intestinal biopsies from controls and patients with celiac disease (CeD). Data are represented as mean

values (n=3). **(D)** Relative RNA expression levels of *LOC339803*, *IL8*, *IL6* and *IL1B* by RT-qPCR in intestinal biopsies from controls (CTRL) and CeD patients (CeD). Data are means ± SEM (n≥9). **(E)** Relative RNA expression values of *LOC339803*, *IL8*, *IL6* and *IL1B* in in tumor tissues from intestinal samples compared to normal tissues from online data from Gent2 database (http://gent2.appex.kr/gent2/). **(F)** Overall survival graph in individuals with high *LOC339803* (*AC016747.3*) expression in the different GI cancers from GEPIA (http://gepia.cancer-pku.cn/detail.php?gene=&clicktag=boxplot). **(G)** Left, quantification of total m^6A levels upon METTL3 overexpression by dot-blot. Right, quantification of *LOC339803* RNA levels by RT-qPCR in METTL3 overexpressing cells (ovMETTL3) in HCT-15 cells. Data are means ± SEM (n=5 independent experiments). p-value determined by Student's t-test. **(H)** Left, quantification of total m^6A levels after cycloleucine treatment by dot-blot. Right, quantification of *LOC339803* RNA levels by RT-qPCR in basal or cycloleucine treated cells in HCT-15 cells. Data are means ± SEM (n=3 independent experiments). p-value determined by Student' t-test.

Figure S5. Schematic representation of *LOC339803* mechanism of action

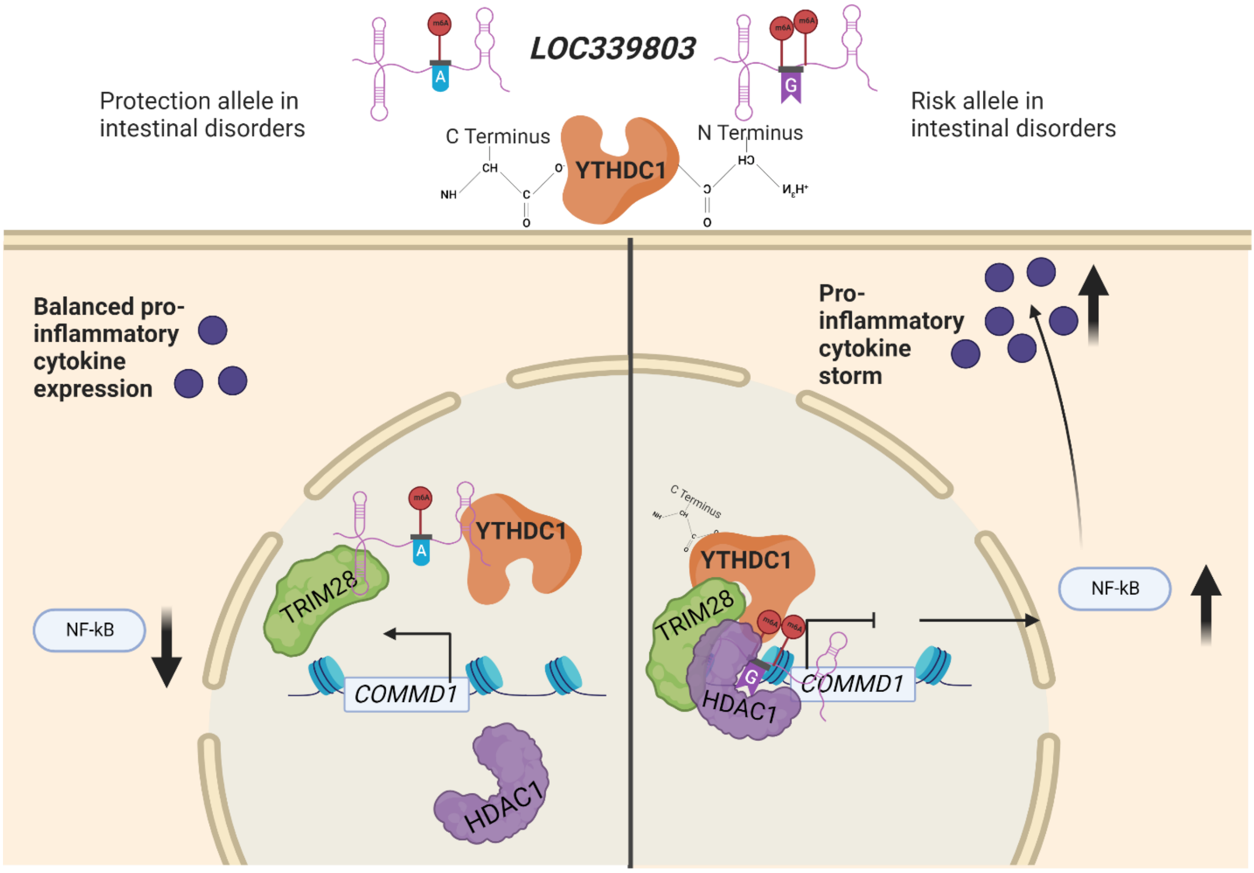

3. SUPPLEMETARY TABLES

Table S1. List of primers and their sequences.

RT-qPCR primers		
**gene name**	**Fw sequence**	**Rv sequence**
LOC339803	TCTTCCTGTGGCCCTCAAAC	TCAACCAAATCGGGAAAGCC
RPLPO	GCAGCATCTACAACCCTGAAG	CACTGGCAACATTGCGGAC
MALAT1	GCTGTGGAGTTCTTAAATAT	TTCTCAATCCTGAAATCCCC
IL8	ACTGAGAGTGATTGAGAGTGGAC	AACCCTCTGCACCCAGTTTTC
IL6	ACTCACCTCTTCAGAACGAATTG	CCATCTTTGGAAGGTTCAGGTTG
IL1B	TTCGACACATGGGATAACGAGG	TTTTTGCTGTGAGTCCCGGAG
lnc13	AAGGATCATTGCAGGGTCTC	GTGGCCAAAAGAAGTCTGAGTC
COMMD1	CTGTTGCCATTATAGAGCTGGAA	GCGTCTTCAGAATTTGGTTGACT
COMMD1 gene body	AGGTTGGCATCTCTGGTTTTAAG	AGTTGGTGCCCTAATGGAAGAG
COMMD1 promoter	GCACAGGCTATTTAGGCACATC	GGTTTGCCACCCTCAAGCTC
COMMD1 DHS	TGTAAGCTGCCAACTCTGACC	TGTGCCTAAATAGCCTGTGC
NFKB	AGAGGCTTCCGATTTCGATATGG	GGATAGGTCTTTCGGCCCTTC
PUS10	AGGTGTGCCAAAAGGTTGAG	CTCTGCTTTCCCATTTCCTG
AHSA2	TCAACGAGTTGAAGCAGGTG	ATCCCTTGTGCTTCACTCCAG
METTL3	TCGAGAGCGAAATTTTTCAAC	GGAGATAGAGAGCCTTCTGAACC
METTL14	GAGTGTGTTTACGAAAATGGGGT	CCGTCTGTGCTACGCTTCA
WTAP	ACTGGCCTAAGAGAGTCTGAAG	GTTGCTAGTCGCATTACAAGGA
FTO	AGATGGAGGGTGTGACAAATG	CATTCTCTCCTCAGGTTCTGG
ALKBH5	CGGCGAAGGCTACACTTACG	CCACCAGCTTTTGGATCACCA
**Primers for plasmid construction**		
**name**	**Fw sequence**	**Rv sequence**
ovLOC	CCGGTACCAGACCTGAAGGCTGCTTCCG	AAGGCCGGCCATCCAAAGTTTCATCCATTT
YTHDC1 C-term	AAATTGGCGCGCCATGAGATTTTTCCTCATAAAG AGTAACAAC	AATTTGGCCGGCCTTATCTTCTATATCGACCTCTC TCCC CTC

LOC339803 T7 promoter sense	GCTAGTGGTGCTAGCCCCGCGAATTAATACGACT ATGCTCATAAACCTGCTCTC	TTTTTGCCTACTCCTTTTTATTC
LOC339803 T7 promoter antisense	GCTAGTGGTGCTAGCCCCGCGAATTAATACGACT TTTTTGCCTACTCCTTTTTATTC	ATGCTCATAAACCTGCTCTC
**CRISPR gRNAs**		
**name**	**Fw sequence**	**Rv sequence**
LOC339803 KO-sg1	CACCGCAATGTGCCATTGTTAGACA	AAACTGTCTAACAATGGCACATTGC
LOC339803 KO-sg2	CACCGTGCGCAGCGGATTTTCCGTT	AAACAACGGAAAATCCGCTGCGCAC
m6A KO_sg1	CACCG TCAGTATCTCTTAAGAACAA	AAACTTGTTCT TAAGAGATAC TGAC
m6A KO_sg2	CACC GTCAATAAGGAGCTGCAATCT	AAACAGATTGCAGCTCCTTATTGAC
**shRNA Oligos for pLKO.1**		
**name**	**Fw sequence**	**Rv sequence**
sh.LOC339803.1	CCGGGAAATGTCAGTATCTCTTAAGCTCGAGCTT AAGAGATACTGACATTTCTTTTTG	AATTCAAAAAGAAATGTCAGTATCTCTTAAGCTC GAGCTTAAGAGATACTGACATTTC
sh.LOC339803.2	CCGGGAGATAAACCACCTGTCATTCCTCGAGGA ATGACAGGTGGTTTATCTCTTTTTG	AATTCAAAAAGAGATAAACCACCTGTCATTCCTC GAGGAATGACAGGTGGTTTATCTC

Table S2. List of *LOC339803* bound proteins in HCT-15 cell lines derived from RIP-MS. **(Separate file)**

4. SUPPLEMENTARY REFERENCES

(1) Nassar, L. R.; Barber, G. P.; Benet-Pagès, A.; Casper, J.; Clawson, H.; Diekhans, M.; Fischer, C.; Gonzalez, J. N.; Hinrichs, A. S.; Lee, B. T.; Lee, C. M.; Muthuraman, P.; Nguy, B.; Pereira, T.; Nejad, P.; Perez, G.; Raney, B. J.; Schmelter, D.; Speir, M. L.; Wick, B. D.; Zweig, A. S.; Haussler, D.; Kuhn, R. M.; Haeussler, M.; Kent, W. J. The UCSC Genome Browser Database: 2023 Update. *Nucleic Acids Res* **2023**, *51* (D1), D1188–D1195. https://doi.org/10.1093/NAR/GKAC1072.

(2) Li, D.; Hsu, S.; Purushotham, D.; Sears, R. L.; Wang, T. WashU Epigenome Browser Update 2019. *Nucleic Acids Res* **2019**, *47* (W1), W158–W165. https://doi.org/10.1093/NAR/GKZ348.

(3) Roadmap Epigenomics Consortium; Kundaje, A.; Meuleman, W.; Ernst, J.; Bilenky, M.; Yen, A.; Heravi-Moussavi, A.; Kheradpour, P.; Zhang, Z.; Wang, J.; Ziller, M. J.; Amin, V.; Whitaker, J. W.; Schultz, M. D.; Ward, L. D.; Sarkar, A.; Quon, G.; Sandstrom, R. S.; Eaton, M. L.; Wu, Y. C.; Pfenning, A. R.; Wang, X.; Claussnitzer, M.; Liu, Y.; Coarfa, C.; Harris, R. A.; Shoresh, N.; Epstein, C. B.; Gjoneska, E.; Leung, D.; Xie, W.; Hawkins, R. D.; Lister, R.; Hong, C.; Gascard, P.; Mungall, A. J.; Moore, R.; Chuah, E.; Tam, A.; Canfield, T. K.; Hansen, R. S.; Kaul, R.; Sabo, P. J.; Bansal, M. S.; Carles, A.; Dixon, J. R.; Farh, K. H.; Feizi, S.; Karlic, R.; Kim, A. R.; Kulkarni, A.; Li, D.; Lowdon, R.; Elliott, G.; Mercer, T. R.; Neph, S. J.; Onuchic, V.; Polak, P.; Rajagopal, N.; Ray, P.; Sallari, R. C.; Siebenthall, K. T.; Sinnott-Armstrong, N. A.; Stevens, M.; Thurman, R. E.; Wu, J.; Zhang, B.; Zhou, X.; Beaudet, A. E.; Boyer, L. A.; De Jager, P. L.; Farnham, P. J.; Fisher, S. J.; Haussler, D.; Jones, S. J. M.; Li, W.; Marra, M. A.; McManus, M. T.; Sunyaev, S.; Thomson, J. A.; Tlsty, T. D.; Tsai, L. H.; Wang, W.; Waterland, R. A.; Zhang, M. Q.; Chadwick, L. H.; Bernstein, B. E.; Costello, J. F.; Ecker, J. R.; Hirst, M.; Meissner, A.; Milosavljevic, A.; Ren, B.; Stamatoyannopoulos, J. A.; Wang, T.; Kellis, M. Integrative Analysis of 111 Reference Human Epigenomes. *Nature* **2015**, *518* (7539). https://doi.org/10.1038/nature14248.

(4) Liu, H.; Wang, H.; Wei, Z.; Zhang, S.; Hua, G.; Zhang, S. W.; Zhang, L.; Gao, S. J.; Meng, J.; Chen, X.; Huang, Y. MeT-DB V2.0: Elucidating Context-Specific Functions of N 6 - Methyl-Adenosine Methyltranscriptome. *Nucleic Acids Res* **2018**, *46* (D1), D281–D287. https://doi.org/10.1093/nar/gkx1080.

(5) Zhou, Y.; Zeng, P.; Li, Y. H.; Zhang, Z.; Cui, Q. SRAMP: Prediction of Mammalian N6-Methyladenosine (M6A) Sites Based on Sequence-Derived Features. *Nucleic Acids Res* **2016**, *44* (10). https://doi.org/10.1093/nar/gkw104.

(6) Mi, H.; Muruganujan, A.; Ebert, D.; Huang, X.; Thomas, P. D. PANTHER Version 14: More Genomes, a New PANTHER GO-Slim and Improvements in Enrichment Analysis Tools. *Nucleic Acids Res* **2019**, *47* (D1). https://doi.org/10.1093/nar/gky1038.

(7) Ashburner, M.; Ball, C. A.; Blake, J. A.; Botstein, D.; Butler, H.; Cherry, J. M.; Davis, A. P.; Dolinski, K.; Dwight, S. S.; Eppig, J. T.; Harris, M. A.; Hill, D. P.; Issel-Tarver, L.; Kasarskis, A.; Lewis, S.; Matese, J. C.; Richardson, J. E.; Ringwald, M.; Rubin, G. M.; Sherlock, G. Gene Ontology: Tool 15ort he Unification of Biology. *Nature Genetics*. 2000. https://doi.org/10.1038/75556.

(8) Carbon, S.; Douglass, E.; Good, B. M.; Unni, D. R.; Harris, N. L.; Mungall, C. J.; Basu, S.; Chisholm, R. L.; Dodson, R. J.; Hartline, E.; Fey, P.; Thomas, P. D.; Albou, L. P.; Ebert, D.; Kesling, M. J.; Mi, H.; Muruganujan, A.; Huang, X.; Mushayahama, T.; LaBonte, S. A.; Siegele, D. A.; Antonazzo, G.; Attrill, H.; Brown, N. H.; Garapati, P.; Marygold, S. J.; Trovisco, V.; dos Santos, G.; Falls, K.; Tabone, C.; Zhou, P.; Goodman, J. L.; Strelets, V. B.; Thurmond, J.; Garmiri, P.; Ishtiaq, R.; Rodríguez-López, M.; Acencio, M. L.; Kuiper, M.; Lægreid, A.; Logie, C.; Lovering, R. C.; Kramarz, B.; Saverimuttu, S. C. C.; Pinheiro, S. M.; Gunn, H.; Su, R.; Thurlow, K. E.; Chibucos, M.; Giglio, M.; Nadendla, S.; Munro, J.; Jackson, R.; Duesbury, M. J.; Del-Toro, N.; Meldal, B. H. M.; Paneerselvam, K.; Perfetto, L.; Porras, P.; Orchard, S.; Shrivastava, A.; Chang, H. Y.; Finn, R. D.; Mitchell, A. L.; Rawlings, N. D.; Richardson, L.; Sangrador-Vegas, A.; Blake, J. A.; Christie, K. R.; Dolan, M. E.; Drabkin, H. J.; Hill, D. P.; Ni, L.; Sitnikov, D. M.; Harris, M. A.; Oliver, S. G.; Rutherford, K.; Wood, V.; Hayles, J.; Bähler, J.; Bolton, E. R.; de Pons, J. L.; Dwinell, M. R.; Hayman, G. T.; Kaldunski, M. L.; Kwitek, A. E.; Laulederkind, S. J. F.; Plasterer, C.; Tutaj, M. A.; Vedi, M.; Wang, S. J.; D'Eustachio, P.; Matthews, L.; Balhoff, J. P.; Aleksander, S. A.; Alexander, M. J.; Cherry, J. M.; Engel, S. R.; Gondwe, F.; Karra, K.; Miyasato, S. R.; Nash, R. S.; Simison, M.; Skrzypek, M. S.; Weng, S.; Wong, E. D.; Feuermann, M.; Gaudet, P.; Morgat, A.; Bakker, E.; Berardini, T. Z.; Reiser, L.; Subramaniam, S.; Huala, E.; Arighi, C. N.; Auchincloss, A.; Axelsen, K.; Argoud-Puy, G.; Bateman, A.; Blatter, M. C.; Boutet, E.; Bowler, E.; Breuza, L.; Bridge, A.; Britto, R.; Bye-A-Jee, H.; Casas, C. C.; Coudert, E.;

Denny, P.; Es-Treicher, A.; Famiglietti, M. L.; Georghiou, G.; Gos, A. N.; Gruaz-Gumowski, N.; Hatton-Ellis, E.; Hulo, C.; Ignatchenko, A.; Jungo, F.; Laiho, K.; Le Mercier, P.; Lieberherr, D.; Lock, A.; Lussi, Y.; MacDougall, A.; Ma-Grane, M.; Martin, M. J.; Masson, P.; Natale, D. A.; Hyka-Nouspikel, N.; Orchard, S.; Pedruzzi, I.; Pourcel, L.; Poux, S.; Pundir, S.; Rivoire, C.; Speretta, E.; Sundaram, S.; Tyagi, N.; Warner, K.; Zaru, R.; Wu, C. H.; Diehl, A. D.; Chan, J. N.; Grove, C.; Lee, R. Y. N.; Muller, H. M.; Raciti, D.; van Auken, K.; Sternberg, P. W.; Berriman, M.; Paulini, M.; Howe, K.; Gao, S.; Wright, A.; Stein, L.; Howe, D. G.; Toro, S.; Westerfield, M.; Jaiswal, P.; Cooper, L.; Elser, J. The Gene Ontology Resource: Enriching a Gold Mine. *Nucleic Acids Res* **2021**, *49* (D1). https://doi.org/10.1093/nar/gkaa1113.

(9) Park, S. J.; Yoon, B. H.; Kim, S. K.; Kim, S. Y. GENT2: An Updated Gene Expression Database for Normal and Tumor Tissues. *BMC Med Genomics* **2019**, *12* (5), 1–8. https://doi.org/10.1186/S12920-019-0514-7/FIGURES/5.

(10) Tang, Z.; Li, C.; Kang, B.; Gao, G.; Li, C.; Zhang, Z. GEPIA: A Web Server for Cancer and Normal Gene Expression Profiling and Interactive Analyses. *Nucleic Acids Res* **2017**, *45* (W1), W98–W102. https://doi.org/10.1093/NAR/GKX247.
